# Adult support during childhood: a retrospective study of trusted adult relationships, sources of personal adult support and their association with childhood resilience resources

**DOI:** 10.1186/s40359-021-00601-x

**Published:** 2021-06-27

**Authors:** Kathryn Ashton, Alisha R. Davies, Karen Hughes, Kat Ford, Andrew Cotter-Roberts, Mark A. Bellis

**Affiliations:** 1 Policy and International Development Directorate, a World Health Organization Collaboration Centre on Investment for Health and Well-Being, Cardiff, CF10 4BZ UK; 2 Knowledge Directorate, Public Health Wales, Cardiff, CF10 4BZ UK; 3 Policy and International Development Directorate, a World Health Organization Collaboration Centre on Investment for Health and Well-Being, Wrexham, LL13 7YP UK; 4 Public Health Collaborating Unit, School of Health Sciences, Bangor University, Wrexham, LL13 7YP UK

**Keywords:** Adverse childhood experiences, Resilience, Trusted adult, Parental programmes

## Abstract

**Background:**

Adverse childhood experiences (ACEs) can affect health and well-being across the life course. Resilience is an individual characteristic that is known to help negate the effect of adversities and potentially transform toxic stress into tolerable stress. Having access to a trusted adult during childhood is critical to helping children build resiliency. Here, we aim to understand the relationship between always having access to trusted adult support and childhood resilience resources, and examine which sources of personal adult support and the number of sources of adult support, best foster childhood resilience.

**Methods:**

A Welsh national cross-sectional retrospective survey (n = 2497), using a stratified random probability sample. Data were collected via face-to-face interviews at participants’ places of residence by trained interviewers. Analyses use chi-square and binary logistic regression methods. Outcome measures were childhood resilience resources, access to an always-available trusted adult, and sources of personal adult support.

**Results:**

Prevalence of access to an always-available trusted adult decreased with increasing number of ACEs from 86.6% of individuals with no ACEs, to 44.4% of those with four or more ACEs (≥ 4). In addition, for those experiencing ≥ 4 ACEs, individuals with no access to a trusted adult were substantially less likely than those with access, to report childhood resilience resources. For example, for individuals with ≥ 4 ACEs, those with access to an always-available trusted adult were 5.6 times more likely to have had supportive friends and 5.7 times more likely to have been given opportunities to develop skills to succeed in life, compared to those with no access to a trusted adult. When looking at sources of personal adult support, resilience levels increased dramatically for those individuals who had either one parent only or two parents as sources of support, in comparison to those without parental support.

**Conclusions:**

Analyses here suggest strong relationships between elements of childhood resilience, constant access to trusted adults and different sources of personal adult support. While the eradication of ACEs remains unlikely, actions to strengthen childhood access to trusted adults may partially ease immediate harms and protect future generations.

**Supplementary Information:**

The online version contains supplementary material available at 10.1186/s40359-021-00601-x.

## Background

A growing body of research shows strong relationships between exposure to adverse childhood experiences (ACEs) before the age of 18 years and their negative impact on health and well-being across the life course [1–3]. ACEs include growing up in a household where a child is subject to violence or neglect, or is exposed to substance misuse, mental illness, parental separation and criminal behaviour leading to incarceration of family members [1]. Experiencing adversity during childhood can lead to chronic stress which impacts on the neurological and physical development of children [4, 5]. The long-term effects of this include the uptake of health-harming behaviours such as alcohol misuse and smoking [1, 3]. Consequently, the effects of lifestyle choices and increased stress experienced by individuals exposed to ACEs, can lead to the development of conditions such as cancer or heart disease during adulthood [6, 7] which in turn can have a major impact on health and public services [8, 9].

Resilience is a developed characteristic of an individual which reflects their ability to transform potentially toxic stress into tolerable stress [10–13]. Developed and nurtured over time as an adaptive outcome not a trait [13, 14], resilience can in turn help negate the effect of adversities and reduce their associated potential harmful and psychological impacts. Resilience has been shown to help to moderate the damaging effects of ACEs, with individuals with higher levels of resilience reporting lower uptake of health-harming behaviours and improved educational outcomes [15–18]. This level of resilience has also been connected to an individual’s degree of risk exposure and the resilience resources available to that individual [19]. Previous research in Wales has indicated that whilst the eradication of ACEs remains unlikely, actions to strengthen resilience assets may help to partially offset their immediate harms [15]. Resilience is commonly used to describe an ability to draw on strengths and assets to cope with adversity [20]. A range of protective factors can help individuals develop resilience which can include: characteristics of the individual, nurturing relationships with their family and adult caregivers, and cohesive social networks and communities [10]. Evidence exists to support the view that resilience does not only come from special individual qualities, but also from everyday normative human resources such as families and communities [21, 22]. The capacity of an individual to adapt their characteristics to develop their resilience depends on their connections to other people and systems external to the individual [14, 18].

A child’s proximity to a trusted adult is critical and can help individuals to build relationships within the family and wider community, and to develop social skills and build trust with others [11]. A supportive parent–child relationship has been suggested as the strongest component in childhood resilience development [23] and plays an enormous role in resilience across the life-span [13]. Resilience literature illustrates the emergence of resilience from adaptive systems in human development which included close relationships with competent and caring adults [12].

Non-parental adult support is also recognised as supportive for childhood resilience, but little is understood about the protection these relationships and assets can offer [11], and whether the number of adults providing support has a critical impact on an individual’s childhood resilience.

Using a national study on ACEs and resilience in Wales we examine how access to an always-available trusted adult during childhood is associated with seven childhood resilience resources, identified within a standard tool for measuring resilience. In addition, we examine whether there is a relationship with the number of adults providing personal support during childhood. Finally, we discuss which combination of adults providing personal support offers a protective factor to build childhood resilience.

## Method

### Survey design

In 2017, a nationally representative household survey was undertaken in Wales, achieving an overall sample size of 2497 Welsh residents aged 18–69 years. Based on other ACE surveys [9], a desired sample size of 2000 individuals was set as previous research has shown this to be an adequate number to perform specific ACE analyses. In addition, a target boost sample of 500 individuals resident in areas with levels of Welsh speakers higher than 40% was also included. Random probability sampling was employed to recruit a representative sample of Welsh residents from across Wales by geography and levels of deprivation using the national postcode address file [24]. The sample was stratified by local Health Board area, and within each Health Board, by deprivation quintile at the Lower Super Output Area (LSOA; geographical areas with a population mean of 1600 [25]). LSOAs were categorised into deprivation quintiles based on their ranking in the Welsh Index of Multiple Deprivation (WIMD).

A letter was sent to each randomly selected household, which provided study information and the option to opt out. Households were visited by trained interviewers (March–June 2017) where householders were presented with a further information sheet on the doorstop which explained the purpose of the study. Individuals were made aware of the confidential and voluntary nature of the study and were provided another opportunity to opt out of the research. All materials were made available in both English and Welsh. Face-to-face interviews were undertaken using computer assisted personal interviewing (CAPI) with sensitive questions self-completed. Only one Welsh resident aged between 18 and 69 years from each household was eligible to participate in the study. If the householder who answered the door were ineligible to participate, the household member with the next birthday was asked to participate. No personal identifiable details were collected from individuals during either the recruitment process or the interview.

In total, 7515 households were selected to participate in the study and were sent a letter of invitation to participate, at which point 11.8% (n = 887) opted out of participating. To achieve the target sample for the study, only 4042 households were contacted on their doorstep. Of these, 645 were ineligible to participate. For example, residents at these households were outside of the desired age range for the study. Of the 3397 eligible households, 888 declined to participate and three interviews could not be completed, which resulted in 2506 individuals participating in the study (completion rate of 73.8%, not including those opting out at the letter stage). For analyses undertaken in this paper, 2497 individuals were included due to nine individuals not completing all required questions (age, sex, ACE questions and CYRM-12 questions).

### Questionnaire

The study questionnaire used established ACE questions taken from the Centers for Disease Control and Prevention short ACE tool [26] and the World Health Organization’s Short Child Maltreatment Questionnaire [27] to retrospectively measure respondents’ exposure to ACEs before the age of 18 years. In total, these questions covered eleven categories of ACE including: physical, verbal and sexual abuse; parental separation; exposure to domestic violence; growing up in a household with mental illness, alcohol abuse, drug abuse or with an individual who was incarcerated; physical neglect and emotional neglect (see Additional file 1: Table S1). As in other studies, individuals were categorised into ACE count groups; having experienced no, one, two to three or four or more ACEs [8].

No validated tool could be identified to retrospectively measure access to resilience resources during childhood. Therefore, questions from the established Child and Youth Resilience Measure (CYRM-12 [28]) were used retrospectively to measure access to childhood resilience resources. For the purpose of these analyses, seven of the 12 childhood resilience resources measures in the CYRM-12 were explored. These were: having a role model, parents/caregivers knew a lot about me, feeling a sense of belonging at school, having supportive friends, knowing where in the community to get help, being given opportunities, and feeling culturally engaged (see Additional file 1: Table S1 for questions and responses included for each resource). Previous research has identified differences in how an always-available trusted adult support is defined or termed within the literature [29]. In line with previous research undertaken in Wales [11], we measured always having access to trusted adult support by the question ‘While you were growing up, before the age of 18, was there an adult in your life who you could trust and talk to about any personal problems’. Responses were dichotomised into those who did or did not have access to always-available trusted adult support during childhood. For the purpose of this study, we chose to exclude those who responded *only sometimes* having access to an adult as we felt it important to focus on those individuals who always had access. Participants were also asked which of a list of adult figures (mother; father; other adult relative; teacher; sports coach; health professional; religious leader; adult neighbour/friend; policeman; social worker) they considered to always be important sources of personal adult support during childhood. Respondents were categorised into six groups; both parents (with other adults), both parents alone, one parent (with other adults), one parent alone, no parents but other adults, and none of those listed. The number of sources of personal adult support was also calculated as the total number of adult figures reported to be an important source of support during their childhood. Demographics collected were: age (categorised into 18–29, 30–39, 40–49, 50–59 and 60–69 years), sex, deprivation quintile, and ethnicity (dichotomised into white and other ethnicities for the purpose of analyses due to the relatively small numbers in each individual non-white ethnic group).

### Statistical analysis

SPSS V.24 was used to undertake the statistical analysis. Chi-square tests were used for initial bivariate analysis of associations between variables. Binary logistic regression methods were also employed to examine independent relationships between access to trusted adults, sources of adult support during childhood, socio-demographics, ACE count and elements of childhood resilience. Confounders accounted for were sex, age (years), deprivation quintile and ethnicity. Adjusted means (95% confidence intervals) for having each individual childhood resilience resource and number of sources of personal adult support were calculated based on best-fit logistic regression models.

## Results

In the final sample used for analyses, 60–69 year olds comprised the largest age group (23.1%) and 18–29 year olds the smallest (17.9%). Females accounted for 54.7% of the sample and individuals residing in the most deprived quintile of the population comprised 15.9%, compared to 21.0% in the least deprived. In total, 96.4% of the sample reported their ethnicity as white. Across the sample, 48.7% of respondents reported having been exposed to at least one ACE, with 13.4% of the sample reporting having experienced four or more ACEs during their childhood (Table 1).

**Table 1:** Logistic regression analysis of ACEs, socio-demographics and their associations with access to an always-available adult during childhood

	Total count		Access to an always-available trusted adult before the age of 18 years	
	N	%	AOR (95% CI)	*p*
*ACE count*				
≥ 4 ACEs	1281	51.3	Ref	***
0 ACEs	478	19.1	9.389 (7.08–12.46)	***
1 ACE	403	16.1	5.995 (4.33–8.30)	***
2–3 ACEs	335	13.4	3.269 (2.40–4.46)	***
*Sex*				
Male	1132	45.3	Ref	
Female	1365	54.7	1.457 (1.89–1.79)	***
*Age (years)*				
60–69	447	17.9	Ref	***
18–29	459	18.4	1.770 (1.27–2.47)	0.469
30–39	501	20.1	1.554 (1.12–2.16)	***
40–49	514	20.6	0.966 (0.72–1.30)	0.100
50–59	576	23.1	1.331 (0.98–1.82)	0.001
*Deprivation quintile*				
1 (least deprived)	468	21.0	Ref	0.043
2	523	25.1	0.676 (0.49–0.94)	**
3	627	19.3	1.019 (0.74–1.41)	0.908
4	481	15.9	0.838 (0.60–1.17)	0.300
5 (most deprived)	398	18.7	0.986 (0.69–1.41)	0.938
*Ethnicity*				
White	2407	96.4	Ref	
Other ethnicities	90	3.6	1.202 (0.67–2.17)	0.540

*Ref* reference category, *ACE*  adverse childhood experience, *AOR* adjusted odds ratio, *CI* confidence interval

***p < 0.001; **p < 0.01

Overall, 77.5% of respondents reported having access to an always-available trusted adult during their childhood (Additional file 1: Table S2). Bivariate analyses identified that the prevalence of access to an always-available trusted adult decreased with increasing ACE count, from 86.6% of individuals with no ACEs to 44.4% amongst those with four or more ACEs (Additional file 1: Table S2). Females were more likely than males to report having constant access to a trusted adult during childhood and access differed across age groups with a lower proportion amongst those aged 40–49 years (Additional file 1: Table S2). After adjusting for socio-demographic confounders, females, those aged 30–39 years and those resident in the least deprived areas were more likely to report always having access to a trusted adult in childhood (Table 1). The strong positive association between having access to a trusted adult and ACEs remained, with respondents who reported no ACEs being 9.4 times more likely to have access to a trusted adult, than those with four or more ACEs (Table 1).

Results suggest always having access to a trusted adult in childhood is strongly related to individuals reporting childhood resilience resources (Tables 2, 3). Across the different ACE count groups, the greatest association was observed for those individuals who had experienced four or more ACEs. For example, 79.1% of individuals reporting four or more ACEs with access to an always-available trusted adult reported that during childhood they felt that they had someone they looked up to, compared to 39.5% of those who had no access to a trusted adult (Table 2). This strong relationship was also observed for resilience resources outside of the family context, for example feeling supported in the community and belonging at school, particularly for those individuals experiencing four or more ACEs (Table 2).

**Table 2:** Bivariate relationship between access to an always-available trusted adult before the age of 18 years, ACEs and childhood resilience resources

	n	Childhood resilience resources (%)						
		Role model	Parents/caregivers knew a lot about me	Belonged at school	Supportive friends	Community help	Given opportunities	Culturally engaged
*No trusted adult support*								
≥ 4 ACEs	185	39.5	34.6	28.1	49.7	26.0	31.0	37.0
2–3 ACEs	116	63.2	50.0	43.5	61.2	36.2	59.1	54.8
1 ACE	87	71.3	71.3	58.6	76.7	44.8	64.4	62.8
0 ACEs	170	81.8	80.6	62.9	75.9	47.6	71.8	68.2
*Yes trusted adult support*								
≥ 4 ACEs	148	79.1	69.6	58.1	84.5	64.2	72.3	68.9
2–3 ACEs	286	88.8	86.4	72.0	88.8	59.1	76.6	80.1
1 ACE	385	95.3	91.7	80.0	95.8	76.0	86.2	86.5
0 ACEs	1102	97.5	95.3	85.9	95.4	78.3	90.7	91.1
*X*^2^		592.433	581.369	376.436	418.867	316.934	409.957	387.716
*p*		***	***	***	***	***	***	***

For a full description of each resilience resource, see Additional file: Table 1

*ACE* adverse childhood experience

***p < 0.001

**Table 3:** Logistic regression analysis of ACEs, always-available trusted adult support in childhood and their associations with childhood resilience resources

Trusted adult support by ACE count		Childhood resilience resources													
		Role model		Parents/caregivers knew a lot about me		Belonged at school		Supportive friends		Community help		Given opportunities		Culturally engaged	
		AOR (95% CI)	*p*	AOR (95% CI)	*p*	AOR (95% CI)	*p*	AOR (95% CI)	*p*	AOR (95% CI)	*p*	AOR (95% CI)	*p*	AOR (95% CI)	*p*
No trusted adult support	≥ 4	Ref	***		***		***		***		***		***		***
	2–3	2.708 (1.67–4.39)	***	1.879 (1.16–3.04)	0.010	1.985 (1.22–3.24)	0.006	1.589 (0.99–2.56)	0.056	1.619 (0.98–2.68)	0.061	3.206 (1.97–5.22)	***	2.087 (1.29–3.37)	0.003
	1	3.979 (2.29–6.92)	***	4.518 (2.58–7.90)	***	3.653 (2.14–6.25)	***	3.204 (1.79–2.74)	***	2.240 (1.31–3.84)	0.003	3.932 (2.29–6.75)	***	2.849 (1.66–4.88)	***
	0	7.548 (4.59–12.41)	***	7.611 (4.66–12.44)	***	4.637 (2.95–7.28)	***	3.214 (2.03–5.10)	***	2.630 (1.68–4.13)	***	5.725 (3.62–9.05)	***	3.986 (2.54–6.67)	***
Yes trusted adult support	≥ 4	5.897 (3.59–9.68)	***	4.437 (2.78–7.08)	***	3.533 (2.23–5.59)	***	5.564 (3.26–9.49)	***	5.199 (3.24–8.34)	***	5.743 (3.56–9.26)	***	4.003 (2.51–6.38)	***
	2–3	12.451 (7.76–19.99)	***	12.098 (7.66–19.10)	***	6.652 (4.40–10.05)	***	7.970 (4.98–12.75	***	4.126 (2.75–6.20)	***	7.290 (4.81–11.05)	***	7.289 (4.78–11.13)	***
	1	31.898 (18.23–55.81)	***	20.407 (12.70–32.80)	***	10.398 (6.92–15.63)	***	23.283 (13.03–41.59)	***	9.114 (6.07–13.68)	***	14.055 (9.17–21.55)	***	11.431 (7.48–17.47)	***
	0	60.698 (37.58–98.05)	***	37.288 (24.64–56.43)	***	15.783 (10.96–22.72)	***	20.103 (13.40–30.17)	***	10.054 (7.01–14.42)	***	21.659 (14.90–31.48)	***	17.601 (12.15–25.49)	***

For a full description of each resilience resource, see Additional file 1: Table S1

*Ref* reference category, *ACE* adverse childhood experience, *AOR* adjusted odds ratio, *CI* confidence interval

***p < 0.001

These relationships remained after accounting for the confounders of sex, deprivation quintile, age and ethnicity (Table 3). In particular for individuals with four or more ACEs, those with access to an always-available trusted adult before the age of 18 years were 5.6 times more likely to feel they had supportive friends and 5.7 times more likely to feel they had been given opportunities to develop skills to succeed in life, compared to those who reported no constant access to a trusted adult (Table 3). Furthermore, logistic analyses showed that the odds of reporting childhood resilience resources increased dramatically for those individuals who had access to an always-available trusted adult and reported no ACEs. For example, individuals with access to an always-available trusted adult who reported no ACEs were 20.1 times more likely to report that they had friends who stood by them during difficult times in childhood compared to those with four or more ACEs without constant access to a trusted adult (Table 3).

Bivariate analyses indicated a positive trend between the number of adults reported as important sources of personal adult support and childhood resilience resources (Table 4). After adjusting for confounders, childhood resilience resources, in particular family resilience elements, had higher odds of being reported by those respondents with more sources of personal adult support. For example, compared to those with no sources of personal adult support, those with one source of adult support were 1.4 times more likely to feel they had supportive friends during childhood, individuals with four or more sources of personal adult support were 15.5 times more likely (Table 5).

**Table 4:** Bivariate association between elements of childhood resilience resources and number of sources of personal adult support

Childhood resilience resources	All		Number of sources of personal adult support (%)						
	%	n	0	1	2	3	≥ 4	*X*^2^ trend	*p*
Role model	87.2	2170	50.4	70.3	90.9	94.2	98.0	404.392	***
Parents/caregivers knew a lot about me	83.8	2089	44.1	67.4	86.9	90.4	96.1	384.604	***
Belonged at school	73.0	1821	37.7	55.3	71.2	78.5	88.9	309.293	***
Supportive friends	87.1	2170	59.8	70.6	89.3	92.2	97.4	294.000	***
Community help	65.9	1641	35.2	45.7	59.8	71.7	84.4	304.088	***
Given opportunities	79.3	1976	48.1	62.7	77.7	86.9	92.0	283.089	***
Culturally engaged	79.6	1983	46.9	58.4	79.5	86.8	94.1	352.537	***

For a full description of each resilience resource, see Additional file 1: Table S1

*ACE *adverse childhood experience

***p < 0.001

**Table 5:** Logistic regression analysis of childhood resilience resources and the number of sources of personal adult support in childhood

	Number of sources of personal adult support				
	0	1	2	3	≥ 4
*Childhood resilience resources*					
Role model					
AOR (95% CI)	Ref	1.816 (1.26–2.62)	5.486 (3.66–8.23)	8.333 (5.17–13.44)	23.320 (13.08–41.59)
*p*	***	***	***	***	***
Parents/caregivers knew a lot about me					
AOR (95% CI)		2.242 (1.55–3.25)	5.035 (3.43–7.39)	6.519 (4.26–9.98)	16.094 (10.08–25.69)
*p*	***	***	***	***	***
Belonged at school					
AOR (95% CI)		1.695 (1.19–2.41)	2.577 (1.84–3.62)	3.674 (2.56–5.27)	7.606 (5.29–10.95)
*p*	***	0.003	***	***	***
Supportive friends					
AOR (95% CI)		1.354 (0.94–1.95)	3.718 (2.51–5.51)	5.046 (3.24–7.86)	15.482 (9.11–26.32)
*p*	***	0.102	***	***	***
Community help					
AOR (95% CI)		1.459 (1.03–2.07)	2.121 (1.52–2.96)	3.535 (2.49–5.03)	7.420 (5.22–10.55)
*p*	***	0.035	***	***	***
Given opportunities					
AOR (95% CI)		1.459 (1.03–2.08)	2.239 (1.58–3.17)	4.178 (2.83–6.16)	6.737 (4.58–9.91)
*p*	***	***	0.036	***	***
Culturally engaged					
AOR (95% CI)		1.389 (0.98–1.98)	3.080 (2.17–4.38)	5.068 (3.43–7.50)	6.743 (4.05–11.23)
*p*	***	0.068	***	***	***

For a full description of each resilience resource, see Additional file 1: Table S1

*Ref* reference category, *AOR* adjusted odds ratio, *CI* confidence interval

***p < 0.001

When looking at types of sources of personal adult support, reported resilience resources increased dramatically for individuals who had either one parent only or two parents as sources of support, in comparison to those without parental support (Additional file 1: Table S3). Interestingly, after accounting for confounders, individuals who considered only adults other than their parents to be a source of personal support were no more likely than individuals who had no sources of personal support to report elements of resilience (Table 6). Individuals with one parent and other supportive adults in their lives had significantly higher odds of reporting all childhood resilience resources than those who only reported having both their parents without other supportive adults (Table 6). For example, individuals with one parent and other supportive adults were 7.4 times more likely than those with no sources of personal adult support to report having a role model, whereas those with both parents only were 5.8 times more likely than those with no sources of support.

**Table 6:** Logistic regression analysis of sources of personal adult support in childhood and their associations with childhood resilience resources

Sources of personal adult support	Childhood resilience resources													
	Role model		Parents/caregivers knew a lot about me		Belonged at school		Supportive friends		Community help		Given opportunities		Culturally engaged	
	AOR (95% CI)	p	AOR (95% CI)	p	AOR (95% CI)	p	AOR (95% CI)	p	AOR (95% CI)	p	AOR (95% CI)	p	AOR (95% CI)	p
None of those listed	Ref	***		***		***		***		***		***		***
No parents but other adults	1.522 (0.94–2.48)	0.091	0.767 (0.47–1.26)	0.298	1.281 (0.79–2.07)	0.311	0.878 (0.55–1.41)	0.591	1.756 (1.09–2.82)	0.200	1.106 (0.69–1.78)	0.677	1.013 (0.63–1.64)	0.958
One parent only	2.218 (1.48–3.32)	***	3.340 (2.22–5.02)	***	1.933 (1.33–2.82)	0.001	1.797 (1.20–2.69)	0.005	1.468 (1.01–2.13)	0.044	1.718 (1.17–2.52)	0.005	1.603 (1.10–2.25)	0.015
One parent with other adults	7.365 (4.66–11.64)	***	7.653 (4.96–11.80)	***	3.954 (2.73–5.84)	***	7.465 (4.51–12.35)	***	3.400 (2.36–4.89)	***	3.481 (2.37–2.11)	***	6.853 (4.48–10.48)	***
Both parents only	5.793 (3.57–9.41)	***	5.711 (3.66–8.92)	***	2.670 (1.84–3.88)	***	3.403 (2.18–5.33)	***	2.082 (1.45–2.99)	***	2.335 (1.59–3.44)	***	2.626 (1.78–3.88)	***
Both parents with other adults	16.599 (10.30–26.75)	***	13.111 (8.67–19.83)	***	5.527 (3.93-0.7.78)	***	8.827 (5.73–13.60)	***	5.192 (3.71–7.26)	***	5.720 (3.99–8.21)	***	7.725 (5.32–11.22)	***

For a full description of each resilience resource, see Additional file 1: Table S1

*Ref*  reference category, *AOR *adjusted odds ratio, *CI *confidence interval

***p < 0.001

## Discussion

The mitigating effect of resilience on the potential long-term impact of ACEs has been identified in previous research [9–11, 15]. The analyses outlined in this paper helps to fill the gap in the evidence on the relationship between trusted adult support and childhood resilience, including demonstrating which sources of personal adult support during childhood have the strongest relationships with resilience resources during childhood.

Consistent with the results of another study [11], results here suggest a substantial proportion of the study population (75.5%) had access to an always-available trusted adult during childhood. This proportion decreased with increasing levels of ACEs and females were more likely than males to report access to such support (Table 1). Analyses in this study highlight that always having access to an always-available trusted adult can act as a facilitator for building childhood resilience resources, both within the family and the wider community, particularly for those individuals with four or more ACEs (Tables 2, 3). When looking at different sources of adult support, analyses showed that having access to a higher number of sources of personal adult support had an association with higher levels of childhood resilience resources (Tables 4, 5). However, further analyses illustrate that in cases where a parent was not reported as a source of personal adult support, levels of resilience resources were not significantly different to those who had no sources of adult support at all, which reinforces the importance of parental support (Table 6). In addition, it is also essential to note the role of personal adult support from individuals outside of the home with analyses showing the positive relationship with childhood resilience, particularly for those individuals with one parent (Table 6).

It is unrealistic that ACEs will be eradicated in the short-term, so this research supports the concept that it is critical to focus on building resilience in children by promoting strong adult–child relationships at home and within in the community [13, 21]. This study provides empirical evidence that demonstrates the importance of a parent as a stable source of support during childhood, and the beneficial impact this can have on levels of childhood resilience. These results support the wider evidence base, which promotes the importance of the first 1000 days of a child’s life and the positive influence of a positive parent–child relationship [30–32]. Early years parental support programmes such as Triple P—Positive Parenting Programme [33] and the Incredible Years programme [34] have been shown to help adults to support children to master key tasks that lay the foundation for future learning, through developing attachments, self-regulatory processes and cognitive and linguistic skills [32]. Programmes such as these can support parents to build key relationships which can potentially reduce ACE exposure and which can in turn help build resilience in childhood. The analyses undertaken within this study show that helping to build child and adult relationships can not only impact on family life, but also open up opportunities for support within the wider community. Individuals in this study who reported parental support were also more likely to report a sense of belonging in their communities, to feel like they had been given opportunities and reported feeling more culturally engaged.

Furthermore, this study emphasises the need for focussed and targeted resilience-building interventions required for those children who face high levels of adversity with no adult or parental support. These interventions have the potential to be tailored to individuals’ personal needs and can offer essential support that may not be readily available within the family environment [35, 36].

There are a number of important limitations to note in this research. As the study undertaken was retrospective in design, the effects of recall bias and potential changes to family patterns and cultures over time should be recognised. Additional limitations to be acknowledged are the potential un-willingness of respondents to report adverse experiences, a potential lack of awareness that the way they were treated was physically abusive or neglectful, and the use of self-reported measures. However, it is important to note that response rates and levels of ACEs reported here were similar to other ACE studies [3, 37]. In addition, the generalisability of the results to populations outside of Wales cannot be established without further research. It was not possible to attribute causation through this study. Relationships between trusted adults, sources of adult support and childhood resilience resources have been identified, and although several confounders have been accounted for through logistic regression, it is recognised there may be other independent factors which could have had an impact on these relationships. For example, the study did not account for how much time was spent with trusted adults during childhood and did not look to analyse in detail the wider sources of support within communities. This study did also not account for whether the supportive adult was also a perpetrator contributing to the ACE score. Finally, due to the limitations around the survey questions used from the Welsh ACE survey, this study did not focus on trusted adults who are temporarily or sporadically part of children’s lives. This is something which could be investigated in further research. This study would benefit from additional research, particularly qualitative in nature to allow relationships to be unpicked to further understand the impact different sources of adult support have on childhood resilience resources. Longitudinal studies would also allow the observation of these relationships in ‘real-time’ and would provide evidence of the long-term impact of different sources of support on health and well-being in later life.

## Conclusions

International evidence has highlighted the detrimental relationship between ACEs and health and well-being in later life [1, 2] and the positive impact building resilience can have on negating the effects of ACEs [11, 12]. Having access to a trusted adult during childhood can have an integral impact on helping to build elements of childhood resilience. Analyses presented in this paper help to fill a gap in the current evidence base on how having access to a trusted adult during childhood can help to build elements of childhood resilience, independent of ACE count. Our analyses also suggest how vital a combination of both parental support and also other adult support is during childhood to help childhood develop resilience both within the family, and the community in which they live. It is widely accepted that the eradication of ACEs is beyond the scope of most communities. However, investment in programmes that help to build elements of childhood resilience may help to counteract some of the impact of ACEs. For example, investing in parenting programmes can help individuals support their children and build strong relationships, providing them with the support to help them build elements of resiliency from an early age. Such programmes can also support the development of the parent–child relationship and support a child’s development throughout their lives, demonstrating strong potential for return on investment in the short-term, and through the long-term gains by helping individuals overcome the potential negative impact of ACEs.

## Supplementary Information

**Additional file 1.** Supplementary Tables.

## Data Availability

Data sets and other materials used in this article are available from the corresponding author on reasonable request.

## Abbreviations

ACE: Adverse childhood experience
AOR: Adjusted odds ratio
CAPI: Computer assisted personal interviewing
CI: Confidence interval
CYRM: Child and Youth Resilience Measure
LSOA: Lower super output area
Ref: Reference category
WIMD: Welsh Index of Multiple Deprivation
