# Supplementary material for: Adult support during childhood: a retrospective study of trusted adult relationships, sources of personal adult support and their association with childhood resilience resources
Source: BMC Psychol. 2021 Jun 27;9:101. doi: 10.1186/s40359-021-00601-x (PMC8237477; doi:10.1186/s40359-021-00601-x)
Supplement: Supplementary file 1 — Additional file 1. Supplementary Tables. [file 40359_2021_601_MOESM1_ESM.docx]

**Additional Table 1. Adverse childhood experience (ACE) and resilience questions with qualifying responses**

|  | | **Question** |  | **Qualifying response** | |
| --- | --- | --- | --- | --- | --- |
| **ACE** | All ACE questions were preceded by the statement “While you were growing up, before the age of 18...” | | | |  |
| *Physical abuse* | | How often did a parent or adult in your home ever hit, beat, kick, or physically hurt you in any way? This does not include gentle smacking for punishment? |  | Once or more than once | |
| *Verbal abuse* | | How often did a parent or adult in your home ever swear at you, insult you, or put you down? |  | More than once | |
| *Sexual abuse* | | How often did anyone at least 5 years older than you (including adults) ever touch you sexually? |  | Once or more than once to any of the questions | |
|  | | How often did anyone at least 5 years older than you (including adults) try to make you touch them sexually? |  |  |  |
|  | | How often did anyone at least 5 years older than you (including adults) force you to have any type of sexual intercourse (oral, anal, or vaginal)? |  |  |  |
| *Physical neglect* | | Did your parent/caregiver for long periods of time not provide you with enough food or drink, clean clothes, or a clean and warm place to live when they could have? |  | Yes | |
| *Emotional neglect* | | Were there times when there was no adult living with you who made you feel loved? |  | More than once | |
| *Parental separation* | | Were your parents ever separated or divorced? |  | Yes | |
| *Domestic violence* | | How often did your parents or adults in your home ever slap, hit, kick, punch, or beat each other up? |  | Once or more than once | |
| *Mental illness* | | Did you live with anyone who was depressed, mentally ill, or suicidal? |  | Yes | |
| *Alcohol abuse* | | Did you live with anyone who was a problem drinker or alcoholic? |  | Yes | |
| *Drug abuse* | | Did you live with anyone who used illegal street drugs or who abused prescription medications? |  | Yes | |
| *Incarceration* | | Did you live with anyone who served time or was sentenced to serve time in a prison or young offenders' institution? |  | Yes | |
|  | |  |  |  | |
| **CHILDHOOD RESILIENCE** | | When you were growing up, during the first 18 years of life, to what extent would the sentences below have described you?  *Response options: Not a lot, a little, somewhat, quite a bit, a lot.* |  |  | |
| *Role model* | | I had people I looked up to |  | Quite a bit / a lot | |
| *Parents / caregivers knew a lot about me* | | My parents / caregivers knew a lot about me |  | Quite a bit / a lot | |
| *Belonged at school* | | I felt I belonged in my school |  | Quite a bit / a lot | |
| *Supportive friends* | | My friends would stand by me during difficult times |  | Quite a bit / a lot | |
| *Community help* | | I knew where to go in my community to get help |  | Quite a bit / a lot | |
| *Given opportunities* | | I had opportunities to develop skills to help succeed in life (like job skills and skills to care for others) |  | Quite a bit / a lot | |
| *Culturally engaged* | | I enjoyed my community's cultures and traditions |  | Quite a bit / a lot | |
| **SOURCES OF PERSONAL ADULT SUPPORT** | | While you were growing up, before the age of 18, was there an adult in your life who you could trust and talk to about any personal problems? |  | Always | |
|  | | During your childhood, which of these adults were important sources of personal support?  *Response Options: Mother, Father, Other adult relative, Teacher, Sports coach, Doctor or nurse (or other health professional), Religious leader, Adult neighbout/friend, Policeman, Social worker.* |  | Always | |

ACE = adverse childhood experience.

**Additional Table 2.** **Bivariate relationship between access to an always-available trusted adult before the age of 18 years and ACEs and socio-demographics**

|  |  | n | % | % **of demographic group reporting access to an always-available trusted adult before the age of 18 years** |
| --- | --- | --- | --- | --- |
|  |  | 2497 |  | 77.5 |
| **ACE count** | 0 ACEs | 1281 | 51.3 | 86.6 |
|  | 1 ACE | 478 | 19.1 | 81.6 |
|  | 2-3 ACEs | 403 | 16.1 | 71.1 |
|  | ≥4 ACEs | 335 | 13.4 | 44.4 |
|  | *X*^2^ |  |  | 283.3 |
|  | p |  |  | *** |
| **Sex** | Male | 1132 | 45.3 | 75.0 |
|  | Female | 1365 | 54.7 | 79.6 |
|  | *X*^2^ |  |  | 7.3 |
|  | p |  |  | 0.0 |
| **Age (years)** | 18-29 | 447 | 17.9 | 80.0 |
|  | 30-39 | 459 | 18.4 | 80.0 |
|  | 40-49 | 501 | 20.1 | 71.7 |
|  | 50-59 | 514 | 20.6 | 78.5 |
|  | 60-69 | 576 | 23.1 | 77.6 |
|  | *X*^2^ |  |  | 13.0 |
|  | p |  |  | 0.0 |
| **Deprivation quintile** | 1 (Least deprived) | 468 | 21.0 | 80.8 |
|  | 2 | 523 | 25.1 | 74.3 |
|  | 3 | 627 | 19.3 | 79.6 |
|  | 4 | 481 | 15.9 | 75.4 |
|  | 5 (Most deprived) | 398 | 15.9 | 73.2 |
|  | *X*^2^ |  |  | 8.7 |
|  | p |  |  | 0.1 |
| **Ethnicity** | White | 2407 | 96.4 | 77.3 |
|  | Other ethnicities | 90 | 3.6 | 82.2 |
|  | *X*^2^ |  |  | 1.199 |
|  | p |  |  | 0.274 |

ACE = adverse childhood experience. ***p<0.001.

**Additional Table 3. Bivariate relationship between sources of personal adult support during childhood and elements of childhood resilience**

|  |  |  |  | **Childhood resilience resources** | | | | | | |
| --- | --- | --- | --- | --- | --- | --- | --- | --- | --- | --- |
|  |  | n |  | Role model | Parents / caregivers knew a lot about me | Belonged at school | Supportive friends | Community help | Given opportunities | Culturally engaged |
|  |  | 2497 |  | 87.1 | 83.8 | 73.0 | 87.1 | 65.9 | 79.3 | 79.6 |
| **Sources of personal adult support** | Both parents with other adults | 1077 |  | 97.3 | 95.2 | 85.8 | 95.7 | 80.1 | 90.9 | 91.8 |
|  | Both parents only | 368 |  | 92.5 | 89.6 | 73.8 | 89.1 | 61.5 | 80.1 | 78.9 |
|  | One parent with other adults | 316 |  | 90.3 | 87.4 | 74.2 | 92.9 | 66.3 | 79.8 | 86.7 |
|  | One parent only | 205 |  | 75.5 | 76.6 | 59.9 | 76.5 | 47.2 | 67.9 | 62.7 |
|  | No parents but other adults | 108 |  | 60.4 | 35.8 | 43.4 | 57.0 | 46.7 | 50.0 | 47.2 |
|  | None of those listed* | 240 |  | 50.4 | 44.1 | 37.7 | 59.8 | 35.1 | 48.1 | 46.9 |
|  | *X*^2^ |  |  | 504.841 | 587.074 | 316.164 | 357.141 | 264.450 | 311.096 | 388.362 |
|  | p |  |  | *** | *** | *** | *** | *** | *** | *** |

ACE = adverse childhood experience. ***p<0.001. For a full description of each resilience resource, see Additional Table 1. *Options included: mother; father; other adult relative; teacher; sports coach; health professional; religious leader; adult neighbour/friend; policeman; social worker.
